# Experience with pyrotinib monotherapy for advanced HER2-positive breast cancer with diffuse hepatic metastases and severe hepatic dysfunction: a case report

**DOI:** 10.1097/RC9.0000000000001067

**Published:** 2026-10-07

**Authors:** Shanshan Jiang, Naiqian Zhang, Tingting Zhang, Dequan Xu, Qingjie Zheng, Duo Xu

**Affiliations:** Jilin Cancer Hospital, Changchun, Jilin, China

**Keywords:** breast cancer, case report, diffuse hepatic metastases, HER2-positive, pyrotinib

## Abstract

**Introduction and importance:**

Hepatic metastases are among the most common distant metastatic sites in advanced breast cancer, occurring in approximately 12–20% of patients with advanced disease. There have been few reports on the treatment of breast cancer patients with severe hepatic dysfunction complicated by hepatic metastases.

**Case presentation:**

This case report presents a 54-year-old female with HER2-positive advanced breast cancer who was diagnosed with diffuse hepatic metastases and severe hepatic dysfunction – an indication for which pyrotinib is contraindicated. Pyrotinib monotherapy was administered as first-line treatment, resulting in rapid relief of clinical symptoms, normalization of liver function, a partial tumor response, and a 12-month first-line progression-free survival. Subsequent antibody-drug conjugate therapy maintained disease control for an additional 13 months.

**Conclusion:**

This case describes a clinical response to pyrotinib monotherapy in a patient with HER2-positive advanced breast cancer, diffuse hepatic metastases, severe hepatic dysfunction, and poor performance status. It provides a descriptive clinical reference for this high-risk population, although further evidence is needed to support broader clinical application.

## Introduction

Although most HER2-positive breast cancer patients receive standardized anti-HER2 adjuvant therapy, hepatic metastases still occur in some cases^[^1,2^]^. Treating such patients with severe hepatic impairment is challenging: reduced hepatic drug clearance leads to unpredictable treatment-related toxicity and suboptimal efficacy. Severe hepatic dysfunction is a formal contraindication to the use of pyrotinib, a small-molecule anti-HER2 tyrosine kinase inhibitor (TKI).

HIGHLIGHTS

- This case report describes pyrotinib monotherapy achieving significant efficacy in a patient with advanced, HER2-positive breast cancer with diffuse hepatic metastases and severe hepatic dysfunction, a condition for which pyrotinib is typically contraindicated.
- Pyrotinib monotherapy was associated with rapid improvement in clinical symptoms and liver function, achieved a partial tumor response, and provided a 12-month first-line progression-free survival.
- This case describes a clinical response to pyrotinib monotherapy in a HER2-positive patient with advanced breast cancer, poor performance status, and severe hepatic impairment; further evidence is required to define its clinical utility in this population.

To our knowledge, no reports have documented pyrotinib monotherapy for HER2-positive advanced breast cancer with diffuse hepatic metastases and severe hepatic dysfunction. We report such a case, in line with the SCARE criteria^[^3^]^, achieving significant clinical efficacy with pyrotinib monotherapy. This study aims to provide a new treatment reference for this patient population, which has limited therapeutic options.

## Case report

A 54-year-old woman with no family history of breast or ovarian cancer and no smoking or drinking habits was admitted with hepatic metastases and jaundice for 6 months. She underwent right breast-conserving surgery with sentinel lymph node biopsy for breast cancer (cT1N0M0) on 20 June 2019. Postoperative pathology confirmed ductal carcinoma *in situ* with an invasive carcinoma (1.0 cm) in the right breast, no sentinel lymph node metastasis (0/4), and immunohistochemistry showing ER-negative, PR-negative, HER-2(3+), and Ki-67 20%, pathologically staged as pT1c pN0 M0 (Stage IA). No adjuvant anti-HER-2 therapy was administered postoperatively. The patient’s primary tumor was only 1.0 cm with no sentinel lymph node metastasis (pT1c pN0 M0, Stage IA). At the time of surgery (2019), national clinical guidelines did not mandate adjuvant anti-HER2 treatment for ultra-low-risk HER2-positive early breast cancer. After comprehensive communication regarding recurrence risks and therapeutic options, the patient and her family signed written informed consent to decline all postoperative systemic therapy.

In July 2021, jaundice led to the detection of hepatic metastases from breast cancer, and no treatment was initiated. On 11 January 2022, the patient presented with worsening jaundice, accompanied by fatigue, weight loss, anorexia, nausea, and abdominal distension, with no history of diabetes or hepatitis.

## Clinical examinations

At admission, physical assessment revealed a distinct icteric facies; the patient could not ambulate independently, with an Eastern Cooperative Oncology Group (ECOG) performance status of 3. Abdominal ultrasonography identified numerous heterogeneous, hypoechoic, solid liver lesions, the maximum of which measured 5.4 × 4.0 cm, accompanied by a 4.3 cm peritoneal effusion (Fig. 1). A subsequent PET-CT scan demonstrated extensive, fused fluorodeoxyglucose-avid diffuse hepatic lesions that could not be accurately measured, together with hepatic cysts, pelvic and peritoneal effusions, and multifocal osseous metastases, consistent with systemic distant tumor spread (Fig. 2). Laboratory investigations confirmed severe combined hepatocellular injury and cholestasis: alanine transaminase (ALT) 193.9 U/L, aspartate transaminase (AST) 547.1 U/L, total bilirubin (TBIL) 139.3 μmol/L, serum albumin (ALB) 27.7 g/L, and alkaline phosphatase (ALP) 955 U/L; direct bilirubin 98.2 μmol/L, gamma-glutamyl transferase 1126 U/L. Baseline coagulation parameters were also abnormal: prothrombin time 15.7 s and international normalized ratio 1.42. Based on these biochemical results, alongside the presence of moderate ascites and normal mental status without hepatic encephalopathy, we completed Child-Pugh scoring, yielding a total score of 12, corresponding to Child-Pugh C severe hepatic impairment.

- **Hepatic biopsy pathology**: malignant adenocarcinoma of breast origin; IHC: ER (−), PR (−), HER-2(3 +), Ki-67 30%, and GCDFP-15 (+). The patient was reclassified as rT1N0M1 (Stage IV).

**Figure 1.**
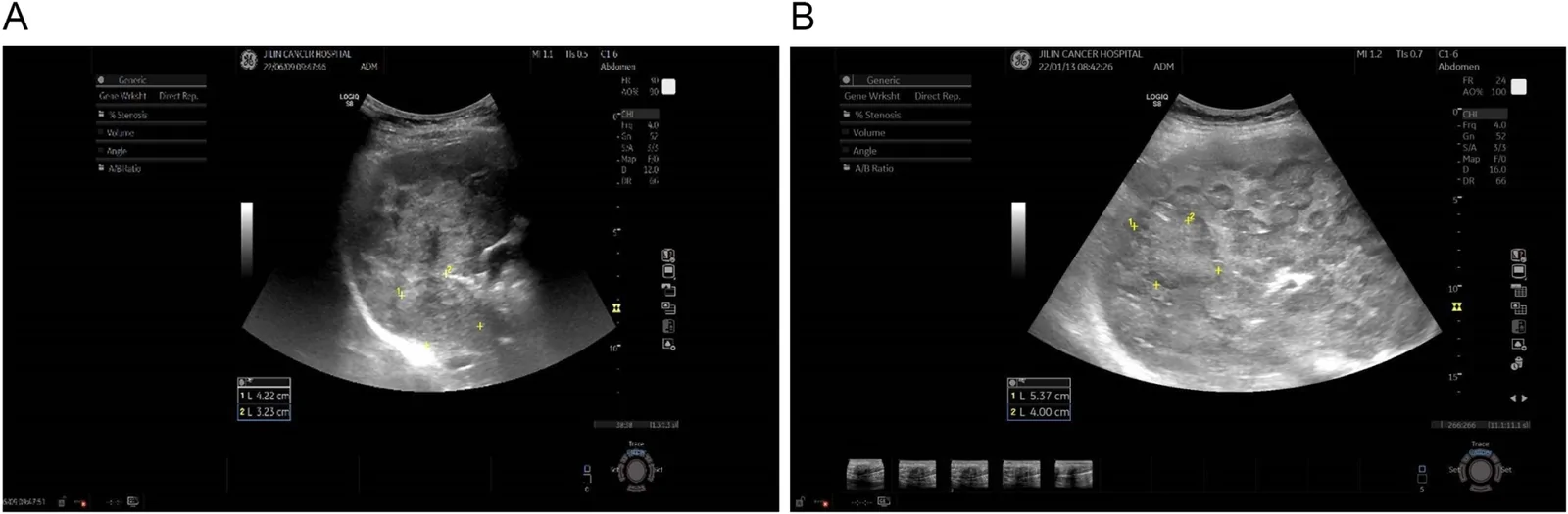
**Ultrasound images of diffuse intrahepatic metastases.** (A) Ultrasound image of the largest lesion, 5.4 × 4.0 cm, in July 2021. (B) Ultrasound image of a metastasis measuring 4.2 × 3.2 cm in June 2022.

**Figure 2.**
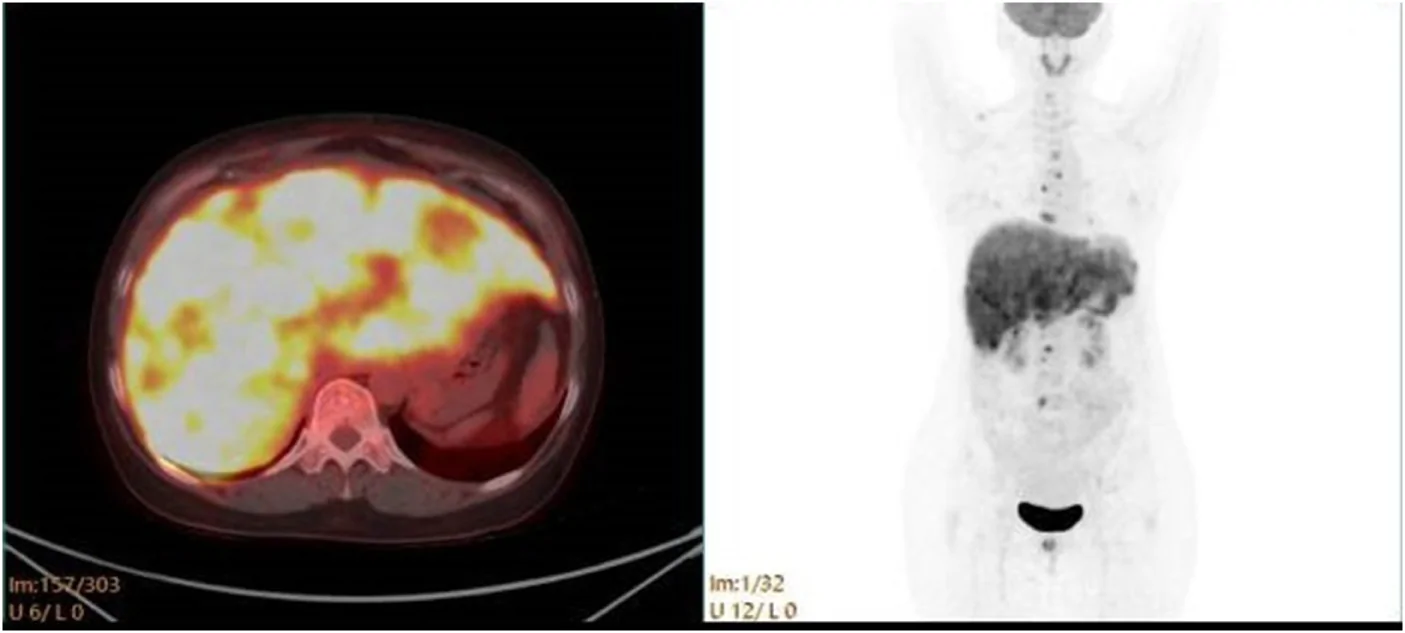
PET-CT images of extensive intrahepatic metastases, July 2021.

## Treatment intervention

A multidisciplinary consultation recommended first-line pyrotinib monotherapy (400 mg orally once daily, 30 minutes after breakfast), considering the patient’s advanced disease, poor performance status, the need for well-tolerated therapy, and limited follow-up during the COVID-19 pandemic. Zoledronic acid (4 mg every 28 days) was administered for bone metastases. Dual antibody–chemotherapy regimens were precluded by Child-Pugh C dysfunction, an ECOG performance status of 3, and the risk of exacerbated hepatotoxicity with intravenous infusion. ADC agents (T-DM1, DS-8201) lack evidence in the setting of acute severe jaundice, while lapatinib and neratinib carry explicit contraindications for moderate to severe hepatic impairment. Pyrotinib offers alternative intestinal and renal clearance pathways, potentially compensating for reduced hepatic metabolism. During the COVID-19 pandemic, intravenous medications were difficult to procure, making oral pyrotinib monotherapy the only feasible first-line option, which was fully endorsed by a multidisciplinary team consultation.

One month post-treatment, jaundice and bone pain resolved, with only Grade 2 diarrhea as a mild adverse event; ECOG performance status improved to 0, and clinical symptoms were significantly ameliorated. Serial liver-function laboratory results after pyrotinib initiation were as follows. Due to COVID-19-associated restrictions, regular short-interval laboratory re-examinations could not be performed. On 9 June 2022: ALT 13.1 U/L, AST 21.5 U/L, ALP 168.3 U/L, ALB 38.9 g/L, and TBIL 13.5 μmol/L. On 8 October 2022: ALT 11.4 U/L, AST 20.0 U/L, ALP 102.1 U/L, ALB 41.5 g/L, and TBIL 16.1 μmol/L. On 27 March 2023: ALT 9.1 U/L, AST 20.0 U/L, ALP 77.9 U/L, ALB 43.1 g/L, and TBIL 9.3 μmol/L. On 30 March 2023: ALT 9.3 U/L, AST 27.2 U/L, ALP 83.8 U/L, ALB 40.7 g/L, and TBIL 10.1 μmol/L. Liver function remained near-normal throughout pyrotinib-based treatment until disease progression (PD)^[^3^]^. The regimen was then switched to pyrotinib plus capecitabine (1.5 g twice daily, 14 days on/7 days off), with trastuzumab added subsequently, but PD occurred after two cycles.

## Follow-up and outcome

First-line PFS was 12 months (PD in February 2023). Second-line treatment with trastuzumab emtansine (T-DM1, 3.6 mg/kg) was initiated but was discontinued due to brain metastasis, with a PFS (PFS2) of 13 months. Third-line treatment with trastuzumab deruxtecan (DS-8201, 5.4 mg/kg) is ongoing with stable disease (Fig. 3).

**Figure 3.**
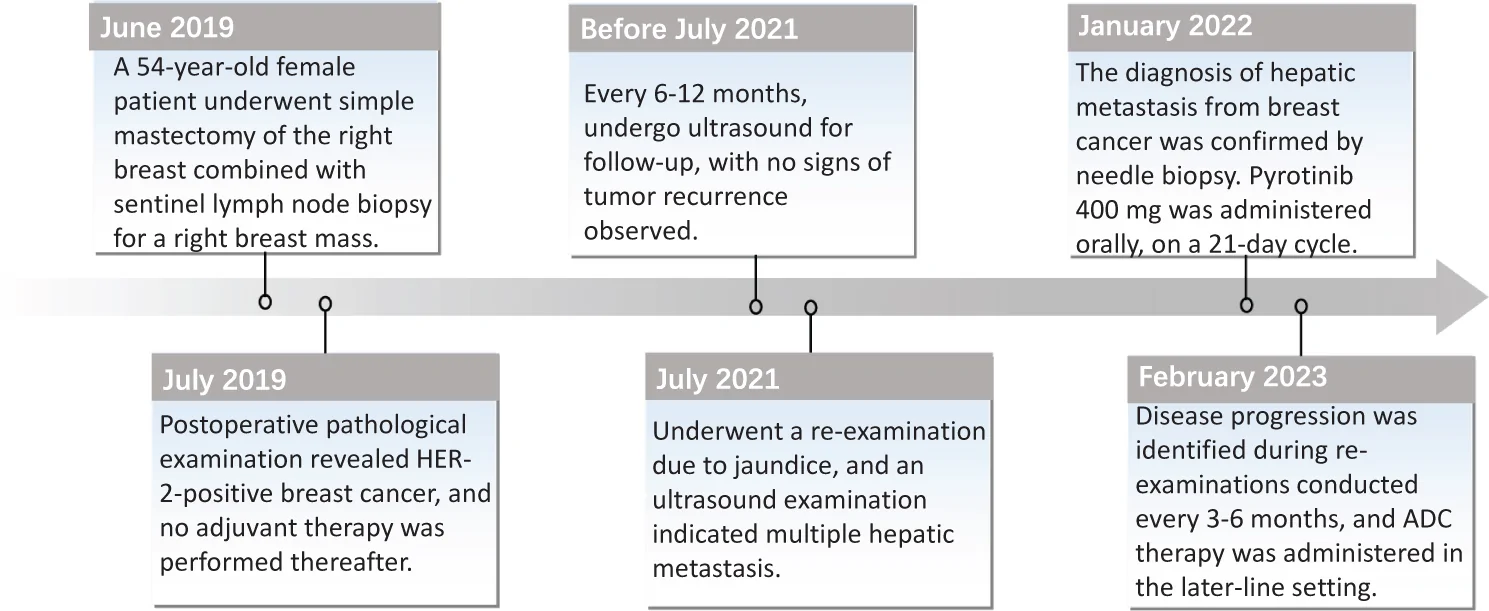
The patient’s timeline of consultations.

## Discussion

This case confirms the efficacy and safety of pyrotinib monotherapy in a patient with HER2-positive advanced breast cancer, diffuse hepatic metastases, severe hepatic dysfunction, an ECOG performance status of 3, and no prior anti-HER2 therapy. Distant metastasis is the leading cause of death in patients with breast cancer, with the liver being the third most common distant metastatic site^[^4^]^; HER2-positive breast cancer carries a higher risk of hepatic metastasis^[^5^]^, with a median survival of only 4–8 months without treatment^[^6^]^.

No standard treatment exists for breast cancer hepatic metastases, and personalized therapy is emphasized^[^7^]^. For metastatic HER-2-positive breast cancer, trastuzumab combined with systemic therapy significantly prolongs overall survival (OS) and progression-free survival (PFS) compared with monotherapy^[^8^]^, and the Food and Drug Administration has approved two anti-HER-2 ADCs (T-DM1 and DS-8201) for this indication^[^9^]^. Pyrotinib was selected based on the phase III PHENIX^[^10^]^ and PHOEBE^[^11^]^ trials, which confirmed its efficacy in second-line advanced HER-2-positive breast cancer; the PHOEBE trial reported a 12.5-month median PFS in the hepatic metastasis subgroup, with significant OS benefits^[^11^]^.

The management of HER2-positive advanced breast cancer with diffuse hepatic metastases and severe liver dysfunction remains a formidable clinical challenge, as this high-risk visceral subtype is routinely excluded from pivotal trials. The CLEOPATRA and PUFFIN studies established first-line trastuzumab plus pertuzumab with taxanes as the standard of care, yielding a median PFS of 18.7 months and OS exceeding 57 months in the overall population, yet both protocols mandated normal baseline hepatic function, thereby precluding patients with overt jaundice or substantial transaminase elevation^[^12,13^]^. Real-world evidence further indicates that in liver-only metastases with normal liver enzymes, dual antibody plus chemotherapy achieves a median PFS of approximately 10.2 months, whereas once ALT/AST exceed three times the upper limit of normal or bilirubin rises, dose reductions become unavoidable, objective response rates drop below 35%, and median PFS shortens to 5.1 months, with chemotherapy often exacerbating hepatic impairment, worsening jaundice and hypoalbuminaemia, and thus being infeasible for patients with poor ECOG performance status and severe cholestatic hepatocellular injury. In this context, the oral small-molecule TKI pyrotinib, used as monotherapy without concurrent cytotoxic agents, offers a potential alternative. In our index case with baseline TBIL of 139.3 μmol/L, AST of 547.1 U/L, and ECOG 3, pyrotinib alone achieved complete recovery of liver function and improvement to ECOG 0 within 1 month, with a first-line PFS of 12 months – superior to historical data for similar patients receiving dual-targeted chemotherapy. However, existing evidence on pyrotinib in this setting is sparse. A multicentre retrospective study^[^14^]^ including 91 HER2-positive advanced breast cancer patients (29 with liver metastases, mostly mild-to-moderate hepatic dysfunction, none with severe jaundice) predominantly used pyrotinib-based combinations, reporting a median PFS of 8.7 months and OS of 15.8 months in the liver metastasis subgroup, with elevated AST in 31% and significantly higher anemia rates; notably, that cohort did not include patients with fulminant hepatic failure, diffuse confluent liver involvement, or ECOG 3, and all received combination therapy, leaving no monotherapy first-line data^[^14^]^. Likewise, the PHENIX and PHOEBE phase III trials excluded moderate-to-severe hepatic impairment, and the pyrotinib label explicitly contraindicates its use in such patients, with no prior prospective study having explored single-agent pyrotinib in jaundiced, diffuse hepatic metastases as first-line treatment^[^10,11^]^. Collectively, these observations underscore the urgent unmet need and the critical knowledge gap regarding pyrotinib monotherapy in this extreme phenotype, while also suggesting that hepatic metastasis per se does not abrogate pyrotinib’s antitumour activity – as reflected by a 72.4% objective response rate and 96.6% disease control rate in the liver metastasis group of the retrospective study – and that toxicities, though including higher risks of anemia and transaminase elevation, remain manageable with supportive care, thereby providing a rationale for carefully selected monotherapy use when standard options are contraindicated.

Pyrotinib is mainly metabolized by CYP3A4/5, and small-molecule TKIs have metabolic flexibility, with alternative clearance pathways (intestinal metabolism and renal excretion) in mild-to-moderate hepatic insufficiency^[^11^]^. Nevertheless, pyrotinib is formally contraindicated for patients with moderate-to-severe hepatic impairment (Child-Pugh B/C), and its use in this patient represents off-label practice with potential unquantified pharmacokinetic and safety risks. This therapeutic decision was made through multidisciplinary tumor board consultation after a comprehensive risk-benefit assessment. Standard combination regimens, including chemotherapy and dual-antibody anti-HER2 therapy, were considered infeasible due to the patient’s ECOG performance status, severe cholestatic hepatocellular injury, and high risk for further hepatic decompensation. Capecitabine was excluded to avoid additional hepatic toxicity. During the COVID-19 pandemic, ensuring continuous intravenous anti-HER2 therapy was difficult. Given the absence of other accessible therapeutic options, oral single-agent pyrotinib was selected after written informed consent was obtained from the patient. It should be emphasized that off-label pyrotinib monotherapy in this contraindicated population carries unknown safety risks, and close laboratory and clinical monitoring is mandatory if similar off-label use is considered in future clinical practice.

This study has several important limitations worthy of clarification. This is merely a retrospective single-case observation without multicenter or large-sample cohort verification, so the therapeutic effect seen in this patient cannot be universally generalized to all HER2-positive breast cancer patients with severe hepatic impairment. Of note, pyrotinib use in this setting constitutes off-label treatment contrary to the official contraindication, and potential safety risks cannot be ruled out based on this single-case report. In addition, we did not implement therapeutic drug monitoring or relevant pharmacokinetic analyses during treatment, thereby failing to clarify the metabolic features of pyrotinib in decompensated liver dysfunction and to provide an objective basis for personalized dose-adjustment schemes for similar high-risk patients. Further real-world studies are urgently needed to evaluate both efficacy and safety in this vulnerable subgroup.

## Conclusion

In summary, this is a single-case observation. It reports a clinical response to pyrotinib monotherapy in a treatment-naive, HER2-positive patient with advanced breast cancer, poor performance status, diffuse hepatic metastases, and severe hepatic dysfunction. Further real-world cohorts and prospective studies are urgently required. Only after validating pyrotinib’s safety and efficacy in this high-risk subgroup can we draw general clinical recommendations.

## Data Availability

Applicable.
